# Exploring Pharmacological Functions of Alternatively Spliced Variants of the Mu Opioid Receptor Gene, *Oprm1*, via Gene-Targeted Animal Models

**DOI:** 10.3390/ijms23063010

**Published:** 2022-03-10

**Authors:** Wenjian Kang, Shan Liu, Jin Xu, Anna Abrimian, Ayma F. Malik, Raymond Chien, Adejuyigbe Adaralegbe, Akwasi Amponsah, Luca Cartegni, John Pintar, Ying-Xian Pan

**Affiliations:** 1 Department of Anesthesiology, Rutgers New Jersey Medical School, Newark, NJ 07103, USA; wj.kang@rutgers.edu (W.K.); sl1848@njms.rutgers.edu (S.L.); jx242@njms.rutgers.edu (J.X.); aa2279@njms.rutgers.edu (A.A.); afm131@gsbs.rutgers.edu (A.F.M.); rc1268@njms.rutgers.edu (R.C.); aa2438@njms.rutgers.edu (A.A.); amponsah@njms.rutgers.edu (A.A.); 2 Department of Chemical Biology, Ernest Mario School of Pharmacy Rutgers University, Piscataway, NJ 08854, USA; luca.cartegni@pharmacy.rutgers.edu; 3 Department of Neuroscience & Cell Biology, Rutgers Robert Wood Johnson Medical School, Piscataway, NJ 08854, USA; pintar@rwjms.rutgers.edu

**Keywords:** opioid, mu opioid receptor, *OPRM1*, alternative splicing, gene targeting, animal model

## Abstract

The mu opioid receptor has a distinct place in the opioid receptor family, since it mediates the actions of most opioids used clinically (e.g., morphine and fentanyl), as well as drugs of abuse (e.g., heroin). The single-copy mu opioid receptor gene, *OPRM1*, goes through extensive alternative pre-mRNA splicing to generate numerous splice variants that are conserved from rodents to humans. These *OPRM1* splice variants can be classified into three structurally distinct types: (1) full-length 7 transmembrane (TM) carboxyl (C)-terminal variants; (2) truncated 6TM variants; and (3) single TM variants. Distinct pharmacological functions of these splice variants have been demonstrated by both in vitro and in vivo studies, particularly by using several unique gene-targeted mouse models. These studies provide new insights into our understanding of the complex actions of mu opioids with regard to *OPRM1* alternative splicing. This review provides an overview of the studies that used these gene-targeted mouse models for exploring the functional importance of *Oprm1* splice variants.

## 1. Introduction

Mu opioids, including those derived from opium, such as morphine, and synthetic ones such as fentanyl and methadone, remain in the mainstream for severe pain management in the clinic despite their side-effects such as tolerance, physical dependence, addiction, constipation, pruritus, and respiratory depression. However, misusing these mu opioids can magnify their side-effects and promote the development of opioid use disorder, a main cause of the worldwide opioid epidemic and opioid overdose deaths, which have been climbing steeply in the United States in recent years [1]. The actions of these mu opioids are primarily mediated through the mu opioid receptor, a G-protein coupled receptor (GPCR) belonging to the class A rhodopsin family. The concept of multiple mu opioid receptors was initially proposed by early pharmacological studies [2,3,4,5,6,7] and further supported by identifying an array of alternatively spliced variants from a single-copy mu opioid receptor gene, *OPRM1* [8,9,10,11,12,13].

**Figure 1:**
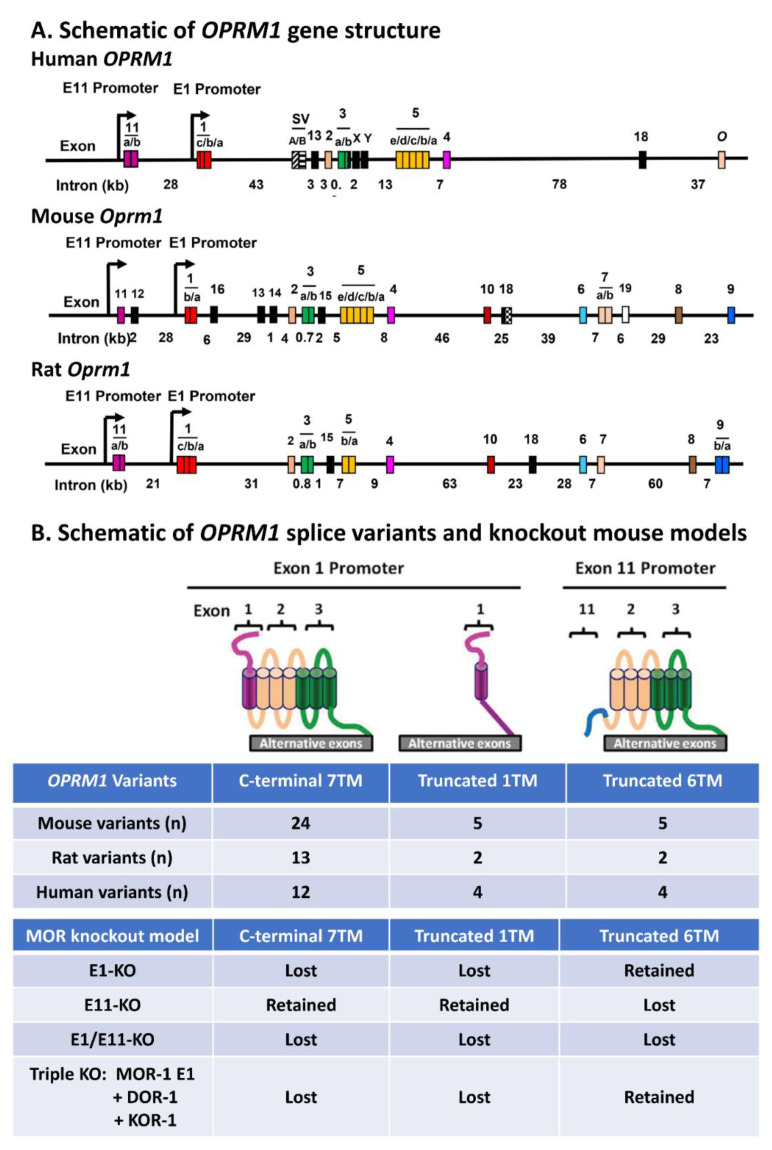
Schematic of the *OPRM1* gene structures, splice variants, and related knockout (KO) mouse models. (**A**) The schematic of the human, mouse, and rat *OPRM1* gene structures adapted from [9]. Exons and introns are shown by colored boxes and horizontal lines, respectively. Promoters are indicated by arrows. Intron sizes are indicated as kilobases (kb). Exons were numbered based on the published data, not by their genomic location, to keep consistent with the literature. The sequence IDs of the human, mouse, and rat *OPRM1* genes in Ensembl are ENSG00000112038, ENSMUSG00000000766, and ENSRNOG00000018191, respectively. Note: the exons in Ensembl or other genome browsers were numbered based on their genomic locations. The exon and intron distances are not drawn to scale. The exon composition of *OPRM1* splice variants and the role of these splice variants were described in previous reviews [8,9,11,12]. (**B**) Schematic of the *OPRM1* splice variants and KO mouse models adapted from [48]. Top panel: Three types of receptor structures, full-length 7TM C-terminal and truncated 1TM and 6TM, that were predicted from their transmembrane domains (TMs). Lower panel of the tables: The upper table indicates the number of each type of the splice variants in the mouse, rat, and human *OPRM1* genes. The bottom table shows four KO mouse models targeting these three types of variants.

The *OPRM1* gene undergoes extensive alternative pre-mRNA splicing, producing three structurally distinct types of splice variants based on transmembrane domains [9]. The first type includes the full-length 7 transmembrane domain (TM) C-terminal variants, and the other two types are the truncated 6TM and 1TM variants, respectively (Figure 1). All three types of the *OPRM1* splice variants are conserved from rodents to humans. The functional relevance of these OPRM1 splice variants in mu opioid pharmacology has been demonstrated by both in vitro and in vivo studies, particularly by using gene-targeted mouse models. This review mainly focuses on the generation of these gene-targeted mouse models, their application in exploring the in vivo functions of *Oprm1* splice variants, and what we have learned from these studies.

## 2. Pharmacology and Molecular Biology of Mu Opioid Receptors

The concept of the mu opioid receptor was first suggested as the M (morphine, mu) receptor by Martin in 1967, simultaneously with the N (nalorphine, possibly the kappa1 receptor) receptor, based on their sensitivities to nalorphine [2]. The pharmacologically defined opioid receptor subtypes, including the mu, delta, and kappa receptors, were then discovered in the 1970s when opioid receptor binding assays were established [3,4,5] and endogenous opioid peptides, including enkephalin, dynorphin, and β-endorphin, were identified [14,15,16,17]. In the early 1990s, molecular cloning of the delta (DOR-1) [18,19], mu (MOR-1) [20,21,22,23], and kappa (KOR-1) [24,25,26] opioid receptors revealed their cDNA and protein sequences, and subsequently their gene structures and chromosomal locations [27,28,29,30].

In the clinic, patients often show different responses to mu opioids’ diverse actions, including analgesia, tolerance, physical dependence, constipation, addiction, and itch. Incomplete cross-tolerance among the mu opioids prompted the development of the clinical practice of opioid rotation, which allows the use of a different mu opioid at lower doses when a patient becomes tolerant to one mu opioid and thus needs much higher doses of that opioid [31,32,33,34,35]. Similar phenomena were observed in animal models [9]. For example, different inbred mouse strains such as C57BL/6J and 129/P3 displayed divergent responses to morphine and heroin in tolerance and physical dependence [36,37,38]. These observations strongly suggest the existence of multiple mu opioid receptors, a hypothesis originally supported by the identification of three mu receptor subtypes, mu_1_, mu_2_, and morphine-6β-glucuronide (M6G), using pharmacological approaches [6,39,40,41,42,43,44,45,46,47]. However, there is only a single copy of the MOR gene, *OPRM1*, raising the question of how the single *OPRM1* gene correlates with multiple mu opioid receptors proposed by clinical and animal observations, as well as early pharmacological studies.

One hypothesis to address this question is that multiple mu opioid receptors can be generated from the single copy of the *OPRM1* gene through alternative splicing, a post-transcriptional event that provides transcriptomic and proteomic diversity in metazoan organisms. Over the past few decades, numerous *OPRM1* splice variants have been isolated by several laboratories [8,9,11], proving that the hypothesis is correct. Although the association of these *OPRM1* splice variants with the mu subtypes defined by the early pharmacological studies remains unclear, identification and characterization of these *OPRM1* splice variants have revolutionized the concept of multiple mu opioid receptors and provided new insight into our understanding of the complex actions of mu opioids [9,11,12].

## 3. Alternative Pre-mRNA Splicing of the Mu Opioid Receptor Gene, *OPRM1*

Soon after MOR-1 cDNA was cloned, the genomic structure of the *OPRM1* gene with four exons and three introns was determined. Identification of additional 5′ and 3′ exons further expanded the length of the *OPRM1* gene to over 200 kb (Figure 1) [9,11]. The *OPRM1* gene utilizes various splicing mechanisms to produce the multiple splice variants that can be classified into three types, the full-length 7TM C-terminal variants, the truncated 6TM variants, and 1TM variants. Expressions of these splice variants are controlled by two distinct promoters, the exon 1 and exon 11 promoters. The overall *OPRM1* genomic structure, exon/intron organization, alternative promoters, and alternative splicing patterns, as well as the identified splice variants, are conserved from rodents to humans, suggesting evolutionary importance of the *OPRM1* gene [9,11,12].

All the full-length 7TM C-terminal variants are encoded by the same exons, 1/2/3, which constitute identical receptors, except for their intracellular C-terminal tails, which are encoded by different exons downstream of exon 3 resulting from alternative 3′ splicing. All the 7TM C-terminal variants display similar binding affinities towards mu opioids since they share the same binding pocket. However, several endogenous opioid peptides such as β-endorphin and dynorphin A show small but significant differences in the *K_i_* values among some 7TM C-terminal variants [49,50,51,52,53,54,55], raising an interesting question of how different C-terminal sequences influence the shared binding pocket. Although the crystal structure of MOR-1 has been revolved [56,57], the N- and C-terminal sequences of the constructs were truncated in order to stabilize the crystal structure. We expect that with the development of high resolution cryo-electron microscopy technology, the structure of the full-length 7TM MOR-1 will be resolved in the near future, which would provide new insights into how the C-terminal tip sequences impact overall receptor structure. Several in vitro studies using cell models have demonstrated the functional relevance of these full-length 7TM C-terminal variants in mu agonist-induced receptor phosphorylation [58], internalization [58,59,60], G protein coupling [50,52,53,54,55,61,62], β-arrestin 2 recruitment [63,64], desensitization [58], and post-endocytic sorting [65]. Yet, the roles of these C-terminal variants in mu opioid pharmacology were mainly revealed by in vivo studies using C-terminal truncation mouse models (see below).

All the 6TM variants except for human MOR-1K and mu_3_ are generated through the exon 11 promoter [9,12]. Splicing from exon 11 to different exons downstream of exon 1 by skipping exon 1 produces a truncated receptor that contains TM2–TM7, encoded by exons 2/3 without exon 1-encoded TM1 and N-terminal sequences. Initially, the role of these 6TM variants was unknown because they did not bind any available radio-labeled opioid ligands when expressed in cell lines. Recently, it was demonstrated that a 6TM variant, mMOR-1G, can dimerize with 7TM mMOR-1 and increase the expression of mMOR-1 at the protein level through a chaperone-like mechanism in the endoplasmic reticulum (ER) of the Tet-Off CHO cell line [66]. However, the pharmacological functions of the 6TM variants were discovered mainly using gene-targeted mouse models (see below).

Alternative splicing from exon 1 to its downstream exons by skipping exon 2 or exons 2/3 or inserting an exon between exons 1 and 2 produces a truncation receptor with only the first TM encoded by exon 1 [9,12]. It is not surprising that these 1TM variants were unable to bind any opioids. However, the functional relevance of these 1TM variants was shown in their ability to enhance expression of 7TM mMOR-1 at the protein level through a similar mechanism as 6TM mMOR-1G [67]. The in vivo function of the 1TM variants was demonstrated to be involved in morphine analgesia by using an antisense approach [67]. An antisense oligonucleotide targeting the 1TM variant mMOR-1S downregulated the full-length 7TM MOR-1 protein and reduced morphine analgesia.

Differential expressions of the *OPRM1* variants were demonstrated at both mRNA and protein levels using regular PCR [51,54,68,69] or qPCR [68,69] and immunohistochemistry approaches [51,70,71,72]. Although the overall expression of the splice variant mRNAs in the whole brain was relatively low, their expressions in individual brain regions were markedly different, suggesting region-specific expression. The expressions of the *OPRM1* variant mRNAs also varied among different inbred mouse strains that had divergent responses to mu opioids [68], indicating the genetic influence on *Oprm1* alternative splicing. Using antibodies raised against exon-specific antigens, the distribution of exon-associated variants in the brain was revealed as region-specific or cell-specific [70,71,72]. Dysregulation of *OPRM1* alternative splicing was observed in morphine-tolerant mice [69], HIV-infected patients [73,74], and the medial prefrontal cortex of human heroin abusers and heroin self-administering rats [75]. Differential expression of the *Oprm1* splice variants was also seen among male and female mice with a C57BL/6 background [76].

## 4. Targeting *Oprm1* in Mice

Soon after the cDNA sequences encoding the mouse MOR-1 were identified in 1994, the partial genomic sequences and chromosomal location of the mouse mu opioid receptor gene (*Oprm1*) were discovered. Although the complete genomic sequences of the whole mouse *Oprm1* gene were not determined until 2000, when the human genome project hit its first milestone, these partial genomic sequences provided sufficient information that quickly led to the generation of multiple *Oprm1*-targeted mouse models using homologous recombination in ES cells. Different targeting strategies were used by several independent laboratories to provide invaluable tools to investigate in vivo functions of the mu opioid receptors (Table 1).

The first-published *Oprm1*-targeted mouse model was generated in Kieffer’s lab [77] by inserting a neo cassette at the end of exon 2, which disrupted the mu opioid receptor at the insertion site at the isoleucine193 position within TM4. These mice showed complete loss of morphine-induced analgesia, physical dependence, and conditioned place preference, indicating that the products of the *Oprm1* gene are the primary in vivo targets of morphine. Targeting exon 2 abolished all 7TM and 6TM variants. However, the mutant mice still expressed a truncated 3TM (TM1/TM2/TM3) receptor and two single TM variants, mMOR-1S and mMOR-1Z, from the exon 1 promoter that was still active (unpublished observation), leading to questions regarding the role of these variants in vivo. Using this mouse model, extensive studies from many different labs have indicated that *Oprm1* is the key gene responsible for the various actions of all mu opioids, including fentanyl, oxycodone, methadone, buprenorphine, and heroin.

Two exon 1 KO mouse models were generated in Uhl’s [78] and Pintar’s [79] labs, respectively, by using a slightly different targeting strategy. In Pintar’s exon 1 KO mouse, a 2.3 kb of the BamHI-HindIII region, including 0.3 kb upstream of the exon 1 translational start codon ATG, the exon 1 coding sequence, and 1.7 kb of the intron, was replaced by a neo cassette. All 7TM and 1TM variants were disrupted. However, the transcripts containing exons 2/3 corresponding to the 6TM variants were expressed in Pintar’s mouse [79] (unpublished observation), providing an important mouse model for investigating the in vivo function of 6TM variants (see below). In Uhl’s exon 1 KO mouse, a 3.2 kb of the BglII-EcoRI region was targeted, which was longer than the 2.3 kb region in Pintar’s mouse model, in particular, with an additional 0.8 kb upstream sequence covering the exon 1 promoter that was deleted. Similar to Pintar’s mouse model, disrupting exon 1 eliminated all 7TM and 1TM variants. However, it is unclear whether 6TM variants were expressed in Uhl’s mouse. Morphine analgesia was lost in both exon 1 KO mouse models, but divergent analgesic responses to M6G and heroin were observed, which is discussed below. Another exon 1 KO mouse was generated in Yu’s lab [80]. Interestingly, this mouse model showed altered hematopoiesis and sexual function, suggesting the role of *Oprm1* in hematopoiesis and reproductive physiology. An exon 2/3 KO mouse was generated in Loh’s lab [81] by replacing exons 2/3 and their adjacent introns with a neo cassette. The analgesic responses to morphine, M6G, and heroin were lost in the exon 2/3-targeted mouse.

A conditional *Oprm1* KO mouse was produced in Kieffer’s lab [84] by floxing exons 2/3 with loxPs, which allowed Cre-mediated recombination at a specific region or time by microinjecting Cre-expressing virus or breeding with a specific Cre mouse. The roles of *Oprm1* at a discrete region on mu opioid-induced motivation [92], naloxone aversion [93], analgesia [94], respiratory depression [95], and reward [96] were demonstrated by using this conditional *Oprm1* KO mouse. Additionally, two KI mouse models in which mCherry and Venus fluorescent proteins were in-frame fused at the end of exon 4-encoded sequences were made in Kieffer’s lab to examine the distribution of MOR-1 at the protein level [82] and biased signaling for mu opioid drugs [83].

Four phosphorylation-deficient mouse models were generated by replacing a serine (S) or threonine (T) with an alanine (A) to investigate the in vivo functions of the critical phosphorylation residues within the intracellular C-terminal sequences. S375 is the primary site of mu agonist-induced phosphorylation both in vitro and in vivo. Initially, an S375A mutant mouse model generated in Schulz’s lab [90] displayed diminished tolerance to high-efficacy mu agonists such as DAMGO, fentanyl, and etonitazene, but not to morphine. Targeting multiple phosphorylation sites of the intracellular C-terminal sequences in the 10ST-A and 11S/T-A mutant mouse models from Schulz’s lab [91] enhanced mu opioid analgesia and diminished tolerance without an effect on mu opioid-induced respiratory depression, constipation, or physical dependence, suggesting a β-arrestin-independent mechanism underlying opioid side-effects. Interestingly, a T394A mutant mouse model from Wang’s lab [89], in which a threonine in the exon 4-encoded C-terminal sequences was replaced by an alanine, showed reduced acute morphine tolerance and increased intravenous heroin self-administration.

Two KI mouse models were generated to study the in vivo role of the A118G single nucleotide polymorphism (SNP), a human *OPRM1* variation associated with drug addiction, in the mouse. In one KI mouse model from Blendy’s lab [87], a mouse A112G allele that corresponds to the human A118G allele was created. The other KI mouse model, from Heilig’s lab [88], was made by replacing the mouse exon 1 with a human exon 1 with either an A or a G allele. Studying these mouse models offered important insight into the functions and mechanisms of this SNP.

To explore in vivo functions of *Oprm1* splice variants, several mouse models were generated, which included an exon 11 KO mouse [85], an exon 1/11 double KO [86], and three C-terminal truncation mouse models in either a C57BL/6J or 129S6/SvEvTac background [63]. Characterizing these mouse models highlighted the importance of *Oprm1* alternative splicing in opioid pharmacology.

## 5. In Vivo Functions of *Oprm1* Alternatively Spliced Variants Using *Oprm1*-Targeted Mouse Models

### 5.1. In Vivo Functions of Exon 11-Associated Oprm1 6TM Variants: Targeting Exons 1 and 11

#### 5.1.1. The Role of 6TM Variants in M6G and Heroin Analgesia

Morphine-6β-glucuronide (M6G) is a major morphine metabolite in humans and contributes to overall morphine analgesia. M6G is over 50-fold more potent than morphine when administered directly into the brain [97]. An M6G receptor subtype was suggested by pharmacological studies using ^3^H-M6G binding [45], analgesic responses of various mu opioids in different mouse strains [44], and sensitivity of 3-O-methylnaltrexone [46], as well as incomplete cross-tolerance to other mu opioids [35,44,98]. Antisense mapping studies further suggested the existence of an M6G receptor mechanism that is associated with exons 2 and 3, but not exons 1 and 4 [47,99]. However, involvement of *Oprm1* 6TM variants in the analgesic action of M6G and heroin was only discovered by using Pintar’s exon 1 KO and Pan’s exon 11 KO mice.

In Pintar’s exon 1 KO mice, morphine analgesia was abolished. However, M6G and heroin analgesia were maintained with slightly reduced potency (over 2-fold higher ED_50_ values than the WT mice) [79]. The M6G analgesia in the exon 1 KO mice was blocked by β-FNA and 3-methoxynaltrexone (3-MeONtx), a selective M6G antagonist, but not by naltrindole, a delta antagonist, nor by NorBNI, a kappa antagonist. Opioid receptor binding assay with ^3^H-M6G in the exon 1 KO mouse brain revealed a binding site with high affinity (the K_D_, 3.7 nM) and low abundance (the B_max_, 12.4 fmol/mg protein). Intracerebroventricular administration of an antisense oligonucleotide against exon 2 significantly reduced the M6G analgesia in the exon 1 KO mice. Furthermore, an exon 2/3 transcript was detected in the exon 1 KO. Together, these results suggested a unique receptor mechanism for M6G and heroin analgesia that is associated with exons 2/3. This receptor mechanism became clearer when the exon 11-associated 6TM variants were discovered and the exon 11 KO mouse was produced.

The mouse *Oprm1* gene produces five exon 11-associated 6TM variants, all of which lack exon 1, which encodes TM1 [98]. Initially, we knew little about the function of the 6TM variants, since they did not bind any radiolabeled opioid ligands when transfected into several cell lines. However, all 6TM variants contain exons 2 and 3, which were expressed in Pintar’s exon 1 KO mouse, raising the possibility that these 6TM variants could be the potential targets responsible for theM6G and heroin analgesia seen in Pintar’s exon 1 KO mouse. This prompted the effort to generate the exon 11 KO mouse model for testing this possibility [85]. In the exon 11 KO mouse, morphine analgesia was unchanged. This is not surprising, since all the 7TM variants under the control of the exon 1 promoter were maintained. However, knocking out all 6TM variants in the exon 11 KO mouse led to marked attenuation of M6G and heroin analgesia, indicating the involvement of the 6TM variants in M6G and heroin analgesia. These 6TM variant transcripts were maintained in Pintar’s exon 1 KO mouse (as above), suggesting that the 6TM variants are indeed responsible for M6G and heroin analgesia seen in this strain. These findings from both exon 11 KO and exon 1 KO mice established the role of the 6TM variants in M6G and heroin analgesia.

On the other hand, M6G and heroin analgesia were lost in Uhl’s exon 1 KO mice [100]. This disparity between Uhl’s and Pintar’s exon 1 KO mice probably was due to the following factors. First, the targeting strategies were different. As mentioned above, a longer upstream region (~0.8 kb) of the coding exon 1 was deleted in Uhl’s mouse, raising questions of whether this disrupted exon 11-associated 6TM variants. It would be interesting to examine if the 6TM variant transcripts could be detected in Uhl’s mouse. Second, different genetic backgrounds have a significant impact on opioid pharmacology, especially in C57BL/6J and 129 mice. Although both strains are inbred, they have several substrains, and even with the same inbred strain, different breeding schemes at different vendors often lead to alteration of their genomic sequences or structures. Uhl’s mouse model was produced by using AB1 ES cells from 129S7/SvEvBrd, while Pintar’s mouse model used CCE ES cells from 129S/SvEv. In addition, the reported data from both labs were obtained by using mice with C57BL/6J/129 mixed backgrounds.

#### 5.1.2. The Role of 6TM Variants in the Analgesic Action of Arylepoxamides, a New Class of Potent and Safer Analgesics

**Figure 2:**
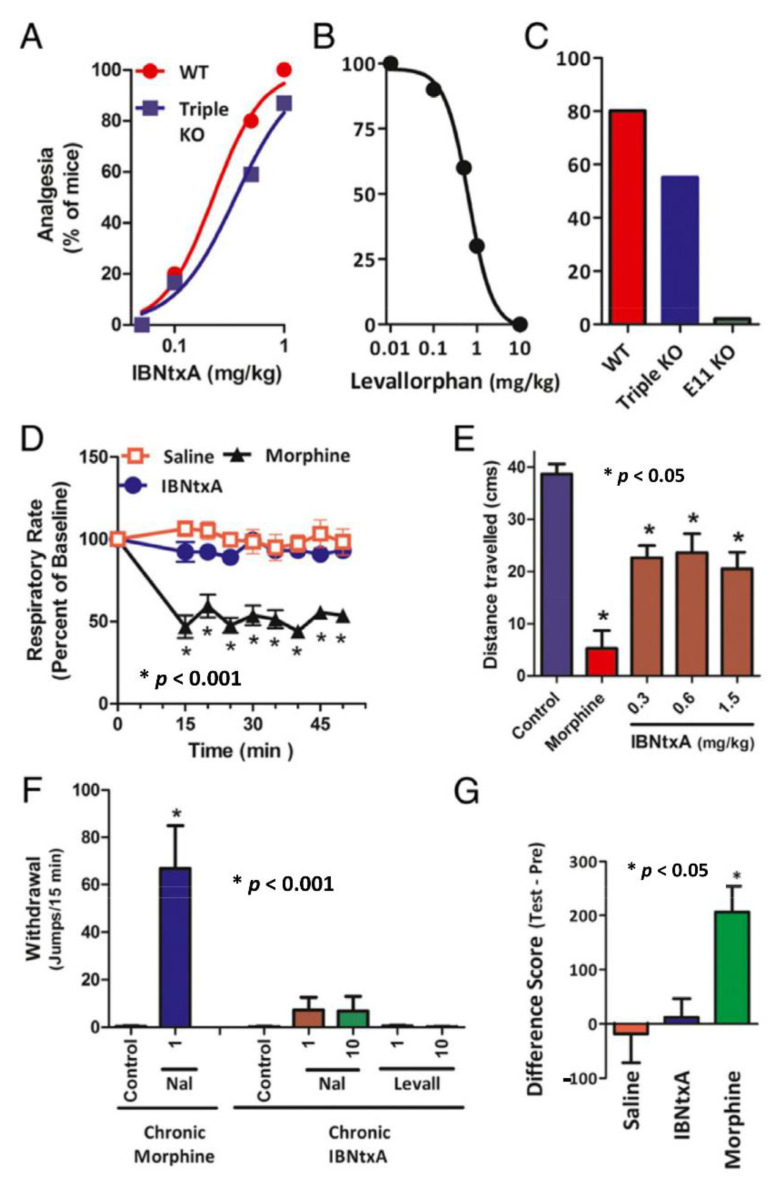
IBNtxA pharmacology (adapted from [101]). (**A**) IBNtxA analgesia. ED_50_ values (and 95% confidence limits) obtained from dose–response curves were 0.22 mg/kg (0.13, 0.32) in WT mice and 0.39 mg/kg (0.15, 0.58) in triple-KO mice by using the radiant heat tail-flick assay. (**B**) Reversal of IBNtxA analgesia by levallorphan. IBNtxA (0.75 mg/kg, s.c.). (**C**) IBNtxA analgesia in KO mice (IBNtxA, 0.5 mg/kg, s.c.). (**D**) Respiratory rate. It was assessed in awake, freely moving CD1 mice that had been administered morphine (20 mg/kg, s.c.), IBNtxA (2.5 mg/kg, s.c.), or saline using the MouseOx pulse oximeter system (Starr Life Sciences). (**E**) Gastrointestinal transit. Morphine (5 mg/kg, s.c.). (**F**) Physical dependence. After being treated with morphine (10 mg/kg, s.c.) or IBNtxA (1 mg/kg, s.c.) for 10 days, mice were challenged with indicated naloxone. The number of jumps during 15 min was counted. (**G**) Conditioned place preference in a two-compartment apparatus with IBNtxA (1 mg/kg) or morphine (10 mg/kg).

A first-generation compound of the arylepoxamide class, 3′-iodobenzoylnaltrexamide (IBNtxA), was synthesized from naltrexone by incorporating an aryl amido group at the 6-position of the morphinan [101,102]. IBNtxA was potent against a spectrum of pain models in animals, particularly for inflammatory and neuropathic pain [103]. However, IBNtxA did not produce several side-effects associated with traditional opiates, including physical dependence, reward, and respiratory depression (Figure 2) [101]. Therefore, IBNtxA serves as a new class of opioid analgesics with high therapeutic potential.

IBNtxA analgesia was abolished in the exon 11 KO mice (Figure 2) [101], suggesting that the exon 11-associated 6TM variants are the molecular targets for IBNtxA. Contrastingly, IBNtxA analgesia was fully maintained in a triple KO mouse model generated in Pintar’s lab. The triple KO mouse model was made by breeding Pintar’s *Oprm1* exon 1 KO mice with his KOR-1 KO and DOR-1 KO mice [101]. Thus, IBNtxA analgesia was solely dependent on the 6TM variants, the only remaining opioid receptors in the triple KO mouse, but independent of 7TM MOR variants, KOR-1 and DOR-1.

**Figure 3:**
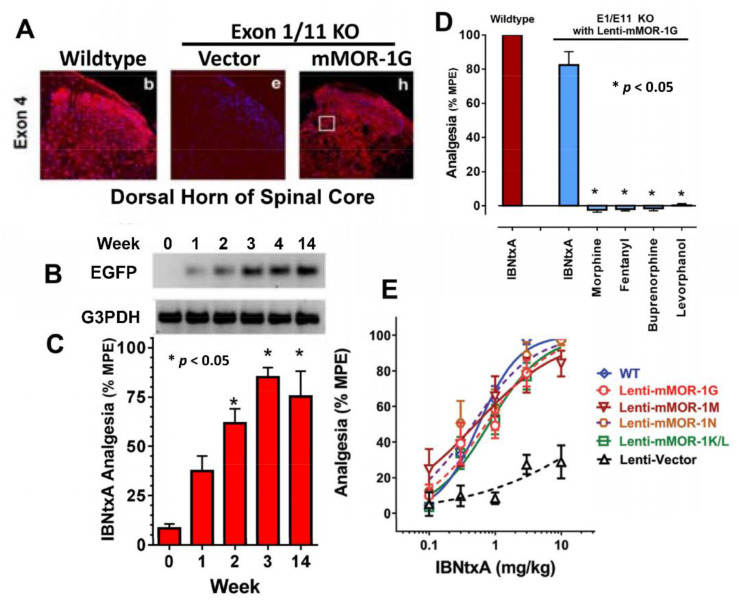
Gain-of-function study using lentivirus expressing the 6TM variants in a double-exon 1/11 KO mouse model. (**A**) Distribution of lentiviral-expressed mMOR-1G in the dorsal horn of the spinal cord with an exon 4 antibody. (**B**) Time course of the spinal EGFP mRNA expression determined by RT-PCR after the lentivirus injection. EGFP was co-expressed in lentivirus as a marker. (**C**) IBNtxA analgesia. (**D**) Opioid analgesia with a single dose of IBNtxA (2.5 mg/kg), morphine (10 mg/kg), fentanyl (0.08 mg/kg), buprenorphine (1 mg/kg), or levorphanol (0.8 mg/kg). (**E**) IBNtxA cumulative dose–response curves. (**A**–**D**) are adapted from [86] and (**E**) is adapted from [48].

To further validate the results from the exon 11 KO and triple KO mice, a gain-of-function study was conducted in a double-exon 1/11 KO mouse model, in which all *Oprm1* splice variants, including all 7TM, 6TM, and 1TM variants, were lost [86]. The analgesic responses for all mu opioids such as morphine, fentanyl, and buprenorphine, as well as IBNtxA, were completely inactive in the double-exon 1/11 KO mouse. Intrathecal delivery of a lentivirus expressing mMOR-1G, a 6TM variant, rescued IBNtxA analgesia, but not analgesia to morphine or other mu opioids. The restoration of IBNtxA analgesia peaked at three weeks after the lentivirus administration and was maintained for at least 33 weeks. The rescued IBNtxA analgesia in the exon 1/11 KO mice had a potency (ED_50_ value) similar to that of WT mice. There are four other 6TM variants in addition to mMOR-1G in mice. To investigate whether the other four 6TM variants, including mMOR-1M, mMOR-1N, mMOR-1K, and mMOR-1L, had a similar function as mMOR-1G, the lentiviruses expressing each individual 6TM variant were administered intrathecally in the exon 1/11 KO mice. All the 6TM variants were able to restore IBNtxA analgesia (Figure 3) [48]. Together, these data illustrated that each of the five 6TM variants are both necessary and essential for IBNtxA analgesia in mice. Therefore, the 6TM variants serve as potential therapeutic targets for developing the new opioid analgesics that are potent but have none of the side-effects associated with traditional opiates. A second generation of arylepoxamides was developed from IBNtxA, and has similar pharmacological profiles as IBNtxA, but is more selective towards the 6TM variants (unpublished data).

A binding site with a unique pharmacological profile in the WT mouse brain was defined by using opioid receptor binding assays with ^125^I-labeled IBNtxA [101]. Since ^125^I-BNtxA can bind to 7TM MOR-1, DOR-1, and KOR-1, the binding assays were performed in the presence of antagonists, including CTAP (7TM MOR-1), naltrindole (DOR-1), and NorBI (KOR-1) to block the binding to these receptors in the WT mice. The direct evidence that this is the binding site for the 6TM variants came from the binding studies using the triple KO mouse brain without these blockers, because they lacked the corresponding receptors [101]. The binding profile in the triple KO mouse was similar to that in the WT mouse, strongly suggesting that the 6TM variants are the targets for ^125^I-BNtxA, since the 6TM variants are the only remaining opioid receptors in the triple KO mouse. However, it remains unknown why the 6TM variants alone, when expressed in cell lines, did not bind ^125^I-BNtxA, raising questions about whether additional molecules or proteins are involved in this unique ^125^I-BNtxA binding site.

#### 5.1.3. Classification of Mu Opioids Based on Alternatively Spliced Oprm1 Variants

The specific exon-targeted mouse models provide useful tools to classify several mu opioids for their analgesic actions based on their association with *Oprm1* splice variants (Table 2). Morphine and methadone analgesia are solely 7TM variant-dependent, as demonstrated by the observation that their analgesia was completely lost in the exon 1-KO mouse [79] but maintained fully in the exon 11-KO mouse [85]. IBNtxA analgesia, on the other hand, is solely 6TM variant-dependent because its analgesia was lost in the exon 11-KO mouse model, and re-expression of 6TM variants in the double exon 1/11-KO mouse model rescued IBNtxA analgesia [48,86]. Several mu opioids, such as buprenorphine and levorphanol, are categorized as both 7TM- and 6TM-dependent opioids based on the following observations. First, their analgesia was inactive in both exon 1-KO and exon 11-KO mice [104]. Second, re-expression of mMOR-1G, a 6TM variant, in the double-exon 1/11-KO mice in which both 6TM and 7TM variants were disrupted failed to restore their analgesia [86]. However, re-expression of mMOR-1G in exon 11-KO mice in which the 7TM variants exist restored their analgesia [105], strongly suggesting that their analgesia is dependent on both 6TM and 7TM variants. M6G and heroin also belong to this category. Intriguingly, a 6TM variant, mMOR-1G, can heterodimerize with 7TM mMOR-1 (based on an in vitro study using a Tet-Off system) to increase the expression of mMOR-1 at the protein level through a chaperone-like mechanism [66], raising the question of whether the analgesic action of these mu opioids dependent on both 6TM and 7TM variants is mediated through the 6TM–7TM heterodimers.

Additionally, several opioid receptor binding studies using brain tissues from the KO mouse models supported the classification of the selected drugs. For example, ^3^H-DAMGO binding was totally abolished in the exon 1-KO mice [79], but maintained in the exon 11-KO mice [85], supporting the dependence of DAMGO analgesia on exon 1-associated 7TM variants. On the other hand, ^125^I-BNtxA binding was lost in the exon 11-KO mice but retained in the triple-KO mice in which the Oprm1 exon 1 was disrupted [101], further suggesting that IBNtxA is 6TM variant-dependent. In addition, the exon 1-KO mice had a low level of ^3^H-M6G binding despite of the loss of ^3^H-DAMGO binding [79], supporting the dependence of M6G analgesia on both 7TM and 6TM variants.

#### 5.1.4. The Role of 6TM Variants in the Analgesic Action of Delta Opioids, Kappa Opioids, and Non-Opioids

The 6TM variants were also shown to involve the analgesia of delta opioids, kappa opioids, and non-opioids by using exon 11-KO and exon 1/11-KO mouse models [107]. Initially, the analgesia of U50,488H, a kappa agonist, was completely lost in the exon 11-KO and exon 1/11-KO mice, but not in exon 1-KO mice, indicating the important role of the 6TM variants in U50,488H analgesia. Delivery of an antisense oligonucleotide targeting exon 11 in WT CD-1 mice significantly reduced U50,488H analgesia, confirming the results from the KO mouse models. Administering the lentivirus expressing a 6TM mMOR-1G variant rescued U50,488H analgesia, not only in exon 11-KO mice, but also in exon 1/11-KO mice, further suggesting that U50,488H analgesia is dependent on 6TM but not 7TM variants. Similar to U50,488H, the analgesia of clonidine, a α2-adernergic agonist, was dependent on 6TM but not 7TM variants. This is supported by the evidence that clonidine analgesia was fully inactive in both exon 11-KO and exon 1/11-KO mice, while the lentivirus expressing mMOR-1G was able to restore its analgesia in exon 1/11-KO mice, and that exon 11-KO mice and the exon 11 antisense oligonucleotide attenuated clonidine analgesia in WT mice.

The analgesia of DPDPE and SNC80, both of which are delta agonists, was diminished in both exon 11-KO and exon 1/11-KO mice [107]. Unlike U50,488H and clonidine, DPDPE and SNC80 analgesia was rescued in exon 11-KO mice in which all 7TM variants were expressed, but not in exon 1/11-KO mice that lacked both 7TM and 6TM variants, implying that both 6TM and 7TM variants are necessary for the delta analgesia. These studies revealed not only the novel functions of the 6TM variants beyond mu opioids, but also the crosstalk or interaction among the GPCRs, either within the opioid receptor system or between opioid receptors and non-opioid receptors. However, the involvement of the 6TM variants in the actions of these delta and kappa opioids and the α2-adernergic drug is only limited to their analgesia, while their other actions, such as SNC80-induced seizure, aversion to U50,488H, and α2-mediated hypolocomotion were not affected in exon 11-KO mice [107].

It remains unknown how 6TM variants are involved in the analgesia of delta, kappa, and α2 drugs. One hypothesis is that heterodimerization, a phenomenon commonly observed among full-length GPCRs [108], of the 6TM variants with another GPCR contributes to the actions. This hypothesis was supported by the observations that the 6TM variants can heterodimerize with DOR-1 and KOR-1 (unpublished observations), although further validation is warranted. Additionally, the heterodimerization between the 6TM variants and the β2-adrenergic receptor was proposed as a mechanism of analgesic synergy seen between 6TM-dependent opioid IBNtxA and β2-adrenergic receptor antagonist [109].

### 5.2. In Vivo Function of Intracellular C-Terminal Tails: Targeting Alternative Exons Downstream of Exon 3

**Figure 4:**
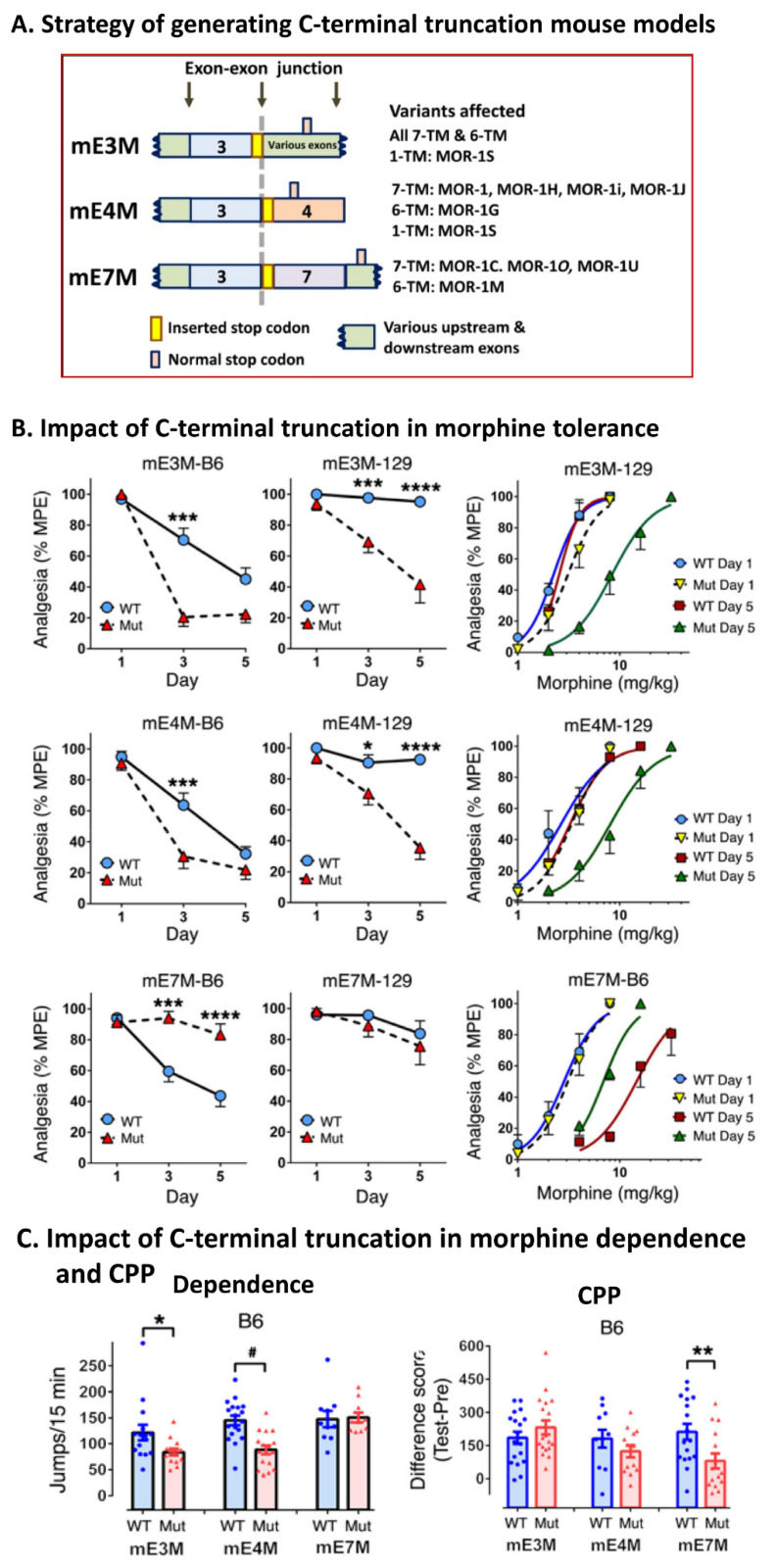
Exploring the roles of alternatively spliced mu opioid receptor C-termini in morphine actions using C-terminal truncation mouse models (adapted from [63]). (**A**) Schematic of the stop codon strategy to generate C-terminal truncation mouse models. Inserted and original stop codons are indicated by yellow and pink bars, respectively. In mE3M, a stop codon was inserted at the end of exon 3. In mE4M and mE7M, a stop codon was inserted at the beginning of exon 4 or exon 7, respectively. (**B**) Impact of C-terminal truncation on morphine tolerance. *: *p* < 0.05; ***: *p* < 0.001; ****: *p* < 0.0001. (**C**) Impact of C-terminal truncation on morphine dependence and CPP. *: *p* < 0.05; **: *p* < 0.01; #: *p* < 0.0001.

Although in vitro studies using cell lines suggested the importance of alternatively spliced C-termini in mu agonist-induced receptor G protein coupling, desensitization, phosphorylation, and post-endocytic sorting, the real questions being raised are about their in vivo functions. Generating C-terminal-targeted mouse models will be ideal to address these questions. All the alternative intracellular C-terminal tails are encoded by different exons downstream of exon 3. Simply knocking out each individual exon would be straightforward. However, this strategy could cause compensatory effects on the expression of the other C-terminal variants, which would make interpretation of the data from the KO mice more difficult. To avoid the potential compensatory effects, a stop codon strategy was used to produce three C-terminal truncation mouse models (Figure 4) [63]. This strategy allows for truncation of the C-terminal sequences at the translational level without affecting overall expression of *Oprm1* transcripts and alternative splicing.

The first C-terminal truncation mouse model, mE3M, was generated by inserting a stop-codon at the end of exon 3 to eliminate all the C-terminal tails encoded by the alternative exons downstream of exon 3 (Figure 4) [63]. The second and third models were mE4M and mE7M, in which a stop-codon was inserted at the beginning of exons 4 or 7, respectively, to truncate the C-terminal tails encoded only by exon 4 or 7 (Figure 4) [63]. As anticipated, the expressions of the overall *Oprm1* transcripts and alternative splicing were not significantly altered except for the lower levels of the exon 7-associated transcripts in mE3M and mE7M mice, which were probably caused by the premature stop codon that was created by the stop codon insertion and regulated by nonsense-mediated mRNA decay, a process that degrades a mRNA with a stop codon located more than 50 nucleotides upstream of the last exon–exon junction [110,111]. Saturation studies in opioid receptor binding assay with ^3^H-naloxone, a reasonable measurement of the expression of opioid receptors at the protein level, revealed that all the C-terminal truncations did not alter the binding affinity (K_D_ value) [63]. However, the expression level (B_max_ value) was significantly reduced in mE3M and mE4M mice with both C57BL/6J and 129SvEv backgrounds, whereas the B_max_ value was not changed in mE7M mice. Although it remains unknown why mE3M and mE4M mice had a lower level of the receptor protein expression while the overall *Oprm1* mRNA level was normal, it is speculated that the loss of the 12 amino acids encoded by exon 4 containing a MOR-1-derived recycling sequence (MRS) [65,112] in mE3M and mE4M mice may contribute to this phenomenon. However, the decrease in the functional receptor proteins did not significantly alter the morphine analgesia potency in mE3M and mE4M mice, suggesting the existence of spare receptors.

#### 5.2.1. The Role of Alternative Intracellular C-Terminal Tails in Morphine Tolerance, Physical Dependence, and Reward

Different inbred mouse strains, particularly C57BL/6J (B6) and 129, have divergent responses to mu opioids [36,37,38]. For example, chronic morphine treatment produces robust tolerance and physical dependence by naloxone-precipitated withdrawal in B6 mice. However, 129 mice do not develop morphine tolerance or physical dependence despite morphine analgesia being equally potent in both strains. Thus, three C-terminal truncation mouse models were generated in both B6 and 129 backgrounds to investigate the role of C-terminal truncation in the different genetic backgrounds [63]. Truncating all the C-termini in mE3M-B6 mice or exon 4-encoded C-termini in mE4M-B6 mice with a B6 background led to the development of morphine tolerance faster than in WT B6 mice (Figure 4). Interestingly, the same truncation in mice with the 129 background made morphine tolerance-resistant 129 mice become morphine tolerance-vulnerable in both mE3M-129 and mE4M-129 models [63].

On the other hand, morphine tolerance was significantly reduced in the exon 7-encoded C-terminal truncation mice with a B6 background (mE7M-B6) [63] (Figure 4), which was opposite to what was seen in mE3M-B6 and mE4M-B6 mice. These results suggest that exon 7-encoded C-terminal tails promote morphine tolerance, while exon 4-encoded C-terminal tails repress it. Administering a vivo-morpholino antisense oligonucleotide targeting the 5′ splicing acceptor site at the intron/exon 7 junction in WT CD-1 mice downregulated exon 7-associated variants and reduced morphine tolerance, mimicking the effect of truncating exon 7 in mE7M-B6 mice [63]. Mu agonist-induced receptor desensitization has been considered as a cellular mechanism of mu opioid tolerance. Among the five brain regions examined, only the hypothalamus and brainstem showed desensitization, measured by the ^35^S-GTPγS binding assay in WT B6 mice [63], suggesting that desensitization in these two regions play an important role in developing morphine tolerance. However, this desensitization in both regions was lost in mE7M-B6 mice [63], implying that exon 7-encoded C-terminal tails contribute to morphine-induced receptor desensitization, which may explain why mE7M-B6 mice were resistant to the development of morphine tolerance.

Three C-terminal truncation mouse models also revealed their unique differences in morphine physical dependence, measured mainly by the number of jumps with naloxone-precipitated withdrawal and morphine reward by CPP [63]. Both mE3M-B6 and mE4M-B6 mice had reduced jumps, while mE7M-B6 mice showed no change. Yet, morphine CPP was significantly reduced in mE7M-B6 mice, but not in mE3M-B6 or mE4M-B6 mice. Together, these results suggest that each C-terminal tail has its unique contribution to morphine actions, highlighting the importance of these C-terminal tails in mu opioid pharmacology.

#### 5.2.2. The Role of Alternative Intracellular C-Terminal Tails in Morphine Locomotion, Catalepsy, and Inhibition of Gastrointestinal Transit

In morphine-induced locomotor responses [63], both mE4M-B6 and mE7M-B6 mice, but not mE3M-B6 mice, had significantly reduced morphine locomotion. Interestingly, all three C-terminal truncation models in B6 backgrounds showed a decrease in morphine-induced catalepsy assays in a dose-dependent and time-dependent manner, suggesting the involvement of these intracellular C-terminal tails in morphine catalepsy. Constipation is one of the major side-effects of morphine and is commonly measured by the inhibition of gastrointestinal transit assay in animal models. All the C-terminal truncation models had no changes in morphine-induced inhibition of GI transit, except for mE4M-B6 mice, which showed reduced inhibition.

#### 5.2.3. Involvement of Exon 7-Associated C-Terminal Variants in β-Arrestin 2-Dependent and -Independent Signaling

Comparison of morphine actions between the mE7M-B6 model and a β-arrestin 2-KO model revealed several similarities [63]. Both models had reduced morphine tolerance and locomotion, no change in morphine physical dependence or inhibition of GI transit, and loss of the desensitized receptor responses in the brainstem, suggesting the functional and physical interaction of exon 7-associated C-terminal tails with β-arrestin 2. The results from in vitro biased signaling assays using ^35^S-GTPγS binding and β-arrestin 2 recruitment assays in PathHuntet cell lines, stably expressing individual exon 7-associated full-length C-terminal splice variants, demonstrated that the exon 7-associated C-terminal variants, particularly mMOR-1O, had greater β-arrestin 2 bias toward several mu agonists than exon 4-associated mMOR-1 [63,64], providing a reasonable explanation for why several morphine responses in mE7M-B6 mice were similar to those in β-arrestin 2-KO mice. Analyzing the 30 amino acids encoded by exon 7, which are conserved from rodents to humans, uncovered a consensus phosphorylation code for high-affinity β-arrestin 2 binding [63], PxPxxE/D, or PxxPxE/D, predicted from homology modeling with the crystal structures of several GPCRs [113]. Disrupting this phosphorylation code in mMOR-1O by mutagenesis markedly reduced DAMGO-induced β-arrestin 2 recruitment (unpublished observation), strongly supporting not only the phosphorylation code mode but also the functional interactions between exon 7-associated variants and β-arrestin 2 seen both in vitro and in vivo.

Nevertheless, several morphine-induced responses, such as analgesia, locomotion, and reward, as well as fentanyl and methadone tolerance in mE7M-B6 mice, were dissimilar [114] (unpublished observation) to those observed in β-arrestin 2-KO mice [115,116,117,118], suggesting that the role of exon 7-encoded C-terminal tails also involves β-arrestin 2-independent mechanisms. Together, the results from the mE7M-B6 mouse model indicated that exon 7-encoded C-terminal tails facilitate the development of several morphine-induced side-effects, including tolerance, locomotion, reward, and catalepsy, without altering morphine analgesia potency. Thus, exon 7-encoded C-terminal tails provide a potential therapeutic target for developing new drugs to minimize these side-effects associated with morphine.

## 6. Conclusions

Extensive alternative splicing of the single-copy *OPRM1* gene generates an array of splice variants, providing molecular diversity and complexity that is exceptional not only within the opioid receptor family but also in GPCRs. Gene-targeting mouse models offer crucial tools to explore the pharmacological functions of these splice variants. Based on exon 1-KO, exon 11-KO, exon 1/11-KO, and triple-KO mouse models, as well as lentiviral rescue studies, we now know that the truncated 6TM variants mediated the analgesic actions of several opioids, including mu drugs such as M6G and heroin, arylepoxamides such as IBNtxA, kappa drug U50,488H, and delta drugs DPDPE and SNC80, as well as the non-opioid α_2_-adernergic drug clonidine. The different C-terminal tails have divergent roles in mu opioid-induced side-effects such as tolerance, physical dependence, and reward, as evidenced by C-terminal truncation mouse models (mE3M, mE4M, and mE7M). More importantly, these studies provide potential therapeutic targets for developing novel analgesics or medications for better pain management. However, these gene-targeted mouse models have a limitation in defining the role of individual variants because many exons are shared by different splice variants, which makes it difficult to create a mouse KO model for a specific splice variant. For example, several full-length 7TM C-terminal variants and one 6TM variant containing exon 7-encoded C-terminal sequences were disrupted in the mE7M mutant mice. Although the exon 7-associated 7TM variants were thought to be involved in the altered morphine actions, it remains unknown which specific variant is responsible. Thus, creating a mouse KO model for a specific splice variant with a conditional setting by using new targeting strategies is essential for studying the pharmacological functions and molecular mechanisms of individual variants in either region-specific or cell-specific fashion. We anticipate seeing such gene-targeted mouse models being developed in the near future, which would be tremendous in advancing our understanding of the roles of *OPRM1* alternative splicing in the complex actions of mu opioids.

## Figures and Tables

**Table 1:** List of the *Oprm1*-targeted mouse models.

Model	Name_Vendor_Stock #	Targeting Site	ES Cell_Strain	Recipient	Lab	Reference
**MOR-KO**	Oprm1^tm1Kff^_Jackson Lab_007559	A neo cassette was inserted in exon 2.	D3/129S2/SvPas	C57BL/6	Kieffer	[77]
**MOR-mCherry**	Oprm1^tm4Kff^_Jackson Lab_029013	mCherry was fused at the end of exon 4.	129svPas-derived	C57BL/6J	Kieffer	[82]
**MOR-Venus**	Oprm1^tm1.1lcs^/KffJ_Jackson Lab_035787	Venus was fused at the end of exon 4.	129svPas-derived	C57BL/6NCrl	Kieffer	[83]
**Oprm1Cre:GFP**	Oprm1^tm1.1(cre/GFP)Rpa^_Jackson Lab_035574	A Cre:GFP/SV40pA cassette was inserted at 5′ of the exon 1 ATG start-codon.	129S6/SvEvTac-derived	C57BL/6NCrl	Kieffer	N/A
**Oprm1fl/fl (conditional KO)**	Oprm1^tm1.1Cgrf^/KffJ_Jackson Lab_030074	Exons 2/3 were floxed with loxPs.	129Sv-derived	C57BL/6J	Kieffer	[84]
**Exon-1 MOR-1 KO**	Oprm1^tm1Uhl^_MGI_363132	A 3.2 kb BglII-EcoRI exon 1-containing region was replaced by a neo cassette.	AB1/129S7/SvEvBrd-Hprt	C57BL/6J	Uhl	[78]
**Exon-1 MOR-1 KO**	N/A	A 2.3 kb BamHI-HindIII exon 1-containing region was replaced by a neo cassette.	CCE/129S/SvEv	C57BL/6	Pintar	[79]
**Exon-1 MOR-1 KO**	N/A	Exon 1 was replaced by a neo cassette.	D3/129S2/SvPas	C57BL/6J	Yu	[80]
**Exons 2/3 MOR-1 KO**	N/A	Exons 2/3 were replaced by a neo cassette.	HM1/129P2/OlaHsd	C57BL/6J	Loh	[81]
**Exon 11 KO**	N/A	Exon 11 was replaced by a LacZ/neo cassette.	E14/129P2/OlaHsd	C57BL/6J	Pan	[85]
**Exons 1/11 double KO**	N/A	Exon 1 and exon 11 were replaced by ZsGreen/SVpA and tdTomamto/BGHpA, respectively.	W4/129S6/SvEvTac	C57BL/6J	Pan	[86]
**mE3M-129**	129S6(B6)-Oprm1^tm3.1Yxp^/Mmmh_ MMRRC_65820	A stop codon was inserted at the end of exon 3 in 129S6/SvEvTac.	W4/129S6/SvEvTac	129S6/SvEvTac	Pan	[63]
**mE3M-B6**	B6(Cg)-Oprm1^tm3.1Yxp^/Mmmh_ MMRRC_625821	A stop codon was inserted at the end of exon 3 in C57BL/6J.	CY2.4/B6(Cg)-Tyr	C57BL/6J	Pan	[63]
**mE4M-129**	129S6(B6)-Oprm1^tm4.1Yxp^/Mmmh_ MMRRC_65823	A stop codon was inserted at the beginning of exon 4 in 129S6/Sv/EvTac.	W4/129S6/SvEvTac	129S6/SvEvTac	Pan	[63]
**mE4M-B6**	B6J.129S6-Oprm1^tm4.1Yxp^/Mmmh_ MMRRC_65822	A stop codon was inserted at the beginning of exon 4 in C57BL/6J.	W4/129S6/SvEvTac	C57BL/6J	Pan	[63]
**mE7M-129**	B6J.129S6-Oprm1^tm2.1Yxp^/Mmmh_ MMRRC_65824	A stop codon was inserted at the beginning of exon 7 in C57BL/6J.	W4/129S6/SvEvTac	129S6/SvEvTac	Pan	[63]
**mE7M-B6**	129S6(B6)-Oprm1^tm2.1Yxp^/Mmmh_ MMRRC_65833	A stop codon was inserted at the beginning of exon 7 in C57BL/6J.	W4/129S6/SvEvTac	C57BL/6J	Pan	[63]
**MOR-KI (A112G)**	Oprm1^tm1jabl^_MGI_3810212	Point mutation of mouse Oprm1 SNP (A112G) that mimicked the human SNP A118G	ES cell/C57BL/6 (Chemicon)	C57BL/6	Blendy	[87]
**Humanized OPRM1-118A** **/OPRM1-118G**	C57BL/6-Oprm1^tm1.1Arte^: 118AC57BL_6-Oprm1^tm2.1Arte^: 118G	Part of mouse exon 1 was replaced by corresponding human exon 1 with the SNP.	ES cell/C57BL/6NTac	C57BL/6	Heilig	[88]
**MOR-KI (T394A)**	Oprm1^tm1.1jbwa^_Jackson Lab_026221	Point mutation at T394	ES cell/129S6/SvEvTac (inGenious Targeting Laboratory)	C57BL/6NTac	Wang	[89]
**MOP KI (S375A)**	Oprm1^tm1Shlz^_MGI_5000465	Point mutation at S375	Bruce 4/B6.Cg-Thy1	C57BL/6J	Schulz	[90]
**MOP KI (10S/T-A)**	Oprm1^tm2.1Shlz^_MGI_6117668	10 serine/threonine phosphorylation sites within the 354–394 region were mutated to alanine.	Bruce 4/B6.Cg-Thy1	C57BL/6J	Schulz	[91]
**MOP KI (11S/T-A)**	Oprm1^tm3.Shlz^_MGI_6117673	11 serine/threonine phosphorylation sites within the 354–383 region were mutated to alanine.	Bruce 4/B6.Cg-Thy1	C57BL/6J	Schulz	[91]

#: Stock number from indicated vendors. MMRRC: Mutant Mouse Resource & Research Center; MGI: Mouse Genome Informatics.

**Table 2:** Classification of mu opioids’ analgesic actions based on alternatively spliced Oprm1 variants.

Classification	Drug	Analgesia					References
		E1-KO	E11-KO	E1/E11-KO	E1/E11-KO + Lenti-6TM	E11-KO + Lenti-6TM	
**7TM variant-** **dependent**	Morphine	Lost	Retained	Lost	N/A	N/A	[79,85]
	Methadone	Lost	Retained	Lost	N/A	N/A	[79,85]
	DAMGO	Lost	Retained	N/A	N/A	N/A	[106]
**7TM + 6TM variant-** **dependent**	Buprenorphine	Lost	Lost	Lost	Not rescued	Rescued	[86,105]
	M6G	Retained but reduced	Reduced	N/A	N/A	N/A	[79,85]
	Heroin	Retained but reduced	Reduced	N/A	N/A	N/A	[79,85]
	DAPP	N/A	Lost	N/A	Not rescued	Rescued	[106]
	IDAPP	N/A	Lost	N/A	Not rescued	Rescued	[106]
	DPDPE	N/A	Lost	N/A	Not rescued	Rescued	[107]
	SNC80	N/A	Lost	N/A	Not rescued	Rescued	[107]
**6TM variant-** **dependent**	IBNtxA	N/A	Lost	Lost	Rescued	N/A	[86,101]
	Ketocyclazocin	N/A	Lost	Lost	Rescued	N/A	[86,101]
	U50,488H	N/A	Lost	Lost	Rescued	Rescued	[107]
	Clonidine	N/A	Lost	Lost	Rescued	Rescued	[107]

## Data Availability

Not applicable.
